# EGFR endocytosis: more than meets the eye

**DOI:** 10.18632/oncotarget.28400

**Published:** 2023-04-10

**Authors:** Aysegul Sapmaz, Ayse Elif Erson-Bensan

**Keywords:** EGFR, SNX3, USP32, endocytosis

## Abstract

Behind the scenes of signaling cascades initiated by activated receptors, endocytosis determines the fate of internalized proteins through degradation in lysosomes or recycling. Over the years, significant progress has been made in understanding the mechanisms of endocytosis and deregulation in disease states. Here we review the role of the EGF-SNX3-EGFR axis in breast cancers with an extended discussion on deregulated EGFR endocytosis in cancer.

## INTRODUCTION

EGFR (Epidermal growth factor receptor) and other cell-surface receptors (e.g., junctional proteins, growth factor receptors, and integrins) have pivotal roles during development and tumorigenesis [1]. Behind the scenes of signaling cascades initiated by activated receptors, endocytosis determines the fate of internalized proteins through degradation in lysosomes or recycling them back to the cell surface or trans-Golgi network (TGN). Over the years, significant progress has improved the understanding of the mechanisms of endocytosis.

Different types of endocytosis (i.e., receptor-mediated endocytosis, clathrin-dependent and -independent endocytosis, caveolar pathway) are generally viewed as a way of receptor attenuation. In contrast, endocytosis also sustains the signaling cascades. Moreover, the roles of endocytic processes in diverse physiological functions, including autophagy, apoptosis, cellular defense, and immune responses, are gaining attention [2, 3]. Hence, endocytic processes may also become oncogenic when deregulated during tumorigenesis, metastasis, and drug resistance in cancers [4]. Here we review the role of the EGF-SNX3-EGFR axis in breast cancers with an extended discussion on canonical and non-canonical functions of endocytic proteins involved in EGFR endocytosis in cancer.

## EGFR ENDOCYTOSIS IN CANCER

High expression of EGFR and its activating mutations in specific cancer types are linked to diverse malignant phenotypes, including stemness, invasion, metastasis, and drug resistance. The underlying mechanisms of EGFR upregulation and oncogenic activity include mutations, amplification of the EGFR gene locus, and defective endocytosis.

Typically, activated and phosphorylated EGFRs undergo internalization and trafficking from the early endosomes to the late endosomes and the lysosomes for degradation. Alternatively, internalized EGFRs are recycled back to the plasma membrane. These processes are heavily dependent on post-translational modifications (e.g., ubiquitination) and are regulated by multiple proteins, including adaptors (e.g., EPS15, Hrs, and STAM), small Rab GTPases, and sorting nexin proteins [5]. Hence deregulation of these players in endocytic processes has significant implications for EGFR activity in cancers.

### Ubiquitination

Ubiquitin serves as a sorting signal during the endocytosis of EGFR while also regulating endocytic proteins [6]. Ligand-activated and autophosphorylated EGFR binds to the E3 ubiquitin ligase, CBL (Casitas B-lineage lymphoma), and gets ubiquitinated, which is essential for clathrin-independent endocytosis (CIE) of EGFR internalization upon high EGF concentrations. CBL directly binds to Py1045 or indirectly via GRB2 adaptor protein to pY1068/pY1086 residues of EGFR. Consequently, EGFR enters the degradation route to the lysosome to prevent continuous receptor activation (reviewed in [7]). In contrast, in cancers, loss of function mutants of CBL have oncogenic activity by enhancing a prolonged receptor tyrosine kinase signaling [8–10].

Ubiquitination of EGFR is also critical for recruiting the adaptor protein, Epidermal Growth Factor Pathway Substrate 15 (EPS15), to coated pits, which is also an essential step for the internalization of EGFR into early endosomes. To get degraded in the lysosome, ubiquitinated EGFR is carried into the intraluminal vesicles (ILVs) of multivesicular buddies (MVBs) via subsequent action of ESCRT-0, I, II, and III complexes, and eventually, the MVBs fuse with the lysosome. Hence, the lack of EGFR ubiquitination allows cancer cells to escape the signal attenuation [11].

Ubiquitination further regulates the function of other proteins involved in endocytic processes. For example, EPS15 is monoubiquitinated by two different E3 ligases [12, 13], which can be reversed by the deubiquitinating enzyme USP9X [14]. When USP9X is depleted, EGFR internalization, trafficking, and turnover are affected [14]. In addition to USP9X, two other DUBs, USP8 and AMSH, bind and deubiquitinate the ESCRT-0 complex to sustain the sorting function of endosomes [15]. Not surprisingly, USP8 hyperactivation due to upregulation and mutations stabilizes numerous oncogenes and activates signaling cascades (e.g., EGFR), thereby contributing to cancer cells’ proliferation and survival [16].

These findings exemplify that the ubiquitination status of the EGFR and other proteins in the endocytic pathway is functionally essential and is balanced by E3 ubiquitin ligases and deubiquitinating enzymes (DUBs). Hence targeting enzymes to alter ubiquitination dynamics could open future perspectives to manipulating EGFR endocytosis and signaling in cancers.

### Small Rab GTPases

The activity of small Rab GTPases depends on their two states: GTP or GDP bound states, representing their active and inactive form, respectively. Activated Rab GTPases then bind to their effector proteins to fulfill their functions. The primary functions of small GTPases and their effectors are associated with membrane identity transitions and cargo transport between various compartments. The first stop of internalized EGFR in this journey is early endosomes, where the sorting occurs, followed by either recycling or degradative routes. The maturation of early to late endosomes is a significant event in the EGFR degradative route, where the switch between small GTPases RAB5 and RAB7 on the endosome surface is critical. Inhibiting this switch, thereby hampering EGFR transport from early to late endosomes, stimulates EGFR recycling back to the plasma membrane. Indeed, the phosphorylation of PRKCD (protein kinase C delta, PKCδ) on the Y374 residue by tyrosine kinase FER (FER tyrosine kinase) is associated with inhibiting EGFR degradation and promoting its recycling via blocking the RAB5-RAB7 switch for endosomal maturation [17]. Interestingly, this specific modification on PKCδ is increased in 25% of triple-negative breast cancer (TNBC) patients, implying a role of endosomal maturation in TNBC pathology [17].

In a recent study that we contributed, spatiotemporal regulation of endocytosis was investigated via modification of the small-RabGTPases [18]. RAB7 was identified as a substrate of USP32, a key deubiquitinase that regulates endocytosis. Mechanistic studies showed that RAB7 effector RILP (Rab interacting lysosomal protein) prefers ubiquitination-deficient RAB7. In contrast, retromer-mediated recycling benefits from RAB7 ubiquitylation, revealing that reversible monoubiquitination of RAB7 regulates distinct functions in endocytosis. As a collective effect of USP32 depletion, EGFR degradation is inhibited, which leads to extended receptor activation. However, an open question is how RAB7 monoubiquitination contributes to USP32 depletion-dependent defects in EGFR degradation and tumorigenesis. Notably, our and others’ work has already linked USP32 to breast and other cancers, highlighting the implications of deregulated USP32 function [19–21].

Additional small GTPases are implicated in tumor formation and progression by deregulating the recycling route of many different cell surface molecules. For instance, RAB25 increases β1 integrin levels and causes subsequent activation of EGFR and upregulation of VEGFA (Vascular Endothelial Growth Factor A), leading to increased Snail expression, epithelial-to-mesenchymal transition, and cancer cell invasiveness [22]. Given their role in endocytic processes, there may be relevant clinical implications related to Rab GTPases and the regulatory mechanisms (including ubiquitination) that modify their functions.

### Non-canonical nuclear functions of endocytic proteins

In addition to these more commonly known functions of endocytosis and endocytosis-related proteins, curiously, non-canonical functions such as nucleocytoplasmic shuttling and transcriptional activity of endocytosis-related proteins are also being described. It appears that endocytic proteins interact with nuclear proteins and modulate the transcription of a set of genes. Indeed, several endocytic proteins have nuclear localization signals (NLSs), or others without an NLS enter the nucleus via protein interactions (Reviewed in [23]). One example is the adapter protein EPS15 which is phosphorylated after EGF stimulation. EPS15, involved in intracellular trafficking, is found mainly in coated pits, but surprisingly, the protein is also present in the nucleus. The mechanism of the nuclear-cytoplasmic shuttling of EPS15 is not entirely known; however, EPS15 in the nucleus is functional as it can positively modulate transcription in a GAL4-based transactivation assay (reviewed in [24]). Similarly, EPN1 (Epsin, EPS15 interactor protein) is generally in the cytosol and is involved in clathrin-mediated endocytosis via direct interactions with EPS15, clathrin, and clathrin adaptor AP-2 (Adaptor Protein Complex 2). EPN1, too, can undergo nucleocytosolic shuttling [25]. Another similar dual-localization is observed for RNF11 (Ring Finger Protein 11), which usually localizes to early endosomes but also appears in the nucleus upon continuous EGF stimulation of EGFR-positive cells. In the nucleus, RNF11 upregulates the transcription of COPII genes (*SEC23B*, *SEC24B*, and *SEC24D*), increasing the efficiency of EGFR transport to the plasma membrane [26]. Accumulating evidence points out that other endocytic proteins and/or adaptors (e.g., ARRB1-2 (Beta arrestin 1-2), CALM (clathrin assembly lymphoid myeloid leukemia protein) shuttle between cytoplasm and nucleus with functions in endocytosis and gene expression (reviewed in [27]). In addition, nucleocytoplasmic shuttling has also been documented for the EGFR family tyrosine kinase receptors themselves (reviewed [28, 29]). However, the biological functions of these nucleocytoplasmic shuttling events are not entirely known.

Overall, it is clear that we need a better understanding of the canonical and non-canonical functions of endocytic processes for normal physiology and diseases, including cancer and other pathologies.

### EGF-SNX3-EGFR axis

Our recent study contributes to understanding the role of deregulated endocytosis in cancer by describing the tumor suppressor role of SNX3 (Sorting Nexin 3) in TNBCs [30]. SNX3 is a member of the recycling retromer complex and is a critical player in the retromer complex.

SNX3 initially attracted our attention as an alternatively polyadenylated transcript in TNBCs [31]. Because SNX3 is involved in endocytosed receptor recycling and half of TNBCs have EGFR overexpression, we investigated whether SNX3 function is important in TNBCs. It turns out SNX3 is a critical player in TNBCs through the EGF-SNX3-EGFR axis.

**Figure 1:**
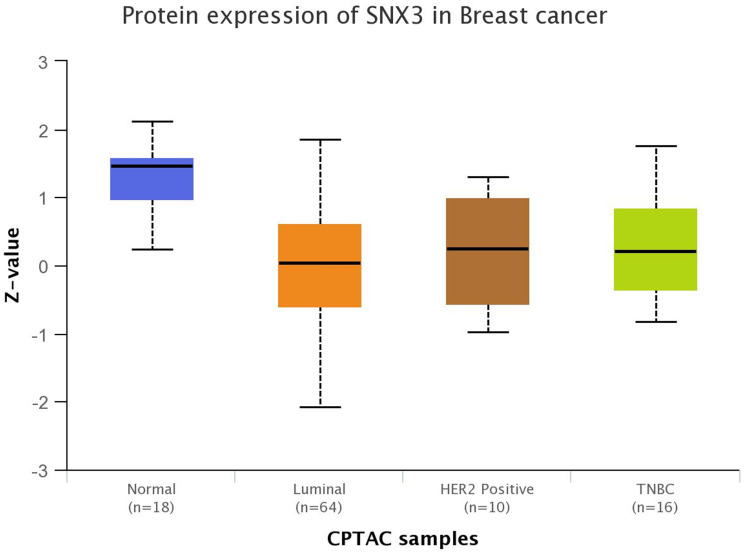
SNX3 protein downregulation in breast tumors. *Z*-values represent standard deviations from the median across samples-data from the UALCAN CPTAC database. SNX3 protein levels are significantly downregulated in Luminal (*p* < 0.0001), HER2 positive (*p* < 0.05), and TNBC tumors (*p* < 0.0001) compared with normal breast tissue (*p* values were calculated by UALCAN).

In EGFR-positive non-tumorigenic mammary cells (MCF10A) and HEK293 cells, EGF treatment causes an immediate upregulation of SNX3 protein levels. The rapid upregulation is initially due to enhanced protein stability, but prolonged EGF stimulation leads to transcriptional upregulation and 3′UTR shortening of *SNX3* [30, 31]. After establishing EGF/EGFR-specific upregulation of SNX3, we investigated how SNX3 might, in turn, regulate EGFR. Given the role of SNX3 in the recycling retromer complex, transient RNAi models of SNX3 downregulation have a temporary reduction in EGFR protein levels. EGF or other ligand-bound EGFRs are typically rapidly endocytosed via clathrin-dependent and independent mechanisms to be recycled back to the plasma membrane from early and sorting endosomes. Alternatively, endocytosed EGFRs are targeted to lysosomes for degradation [3]. Hence, it is intuitive to expect EGFR downregulation when a retromer complex protein is silenced. Indeed that was the case when we transiently silenced SNX3. However, we had an unexpected EGFR protein overexpression when SNX3 was silenced long-term. An unclear compensatory mechanism in SNX3-silenced TNBC cells upregulates *EGFR* transcription and causes EGFR protein overexpression. As a result, TNBC cells become more proliferative, more migratory, and invasive, forming larger primary tumors and causing metastasis in syngeneic models. These findings are also clinically relevant as low *SNX3*, and high *EGFR* mRNA levels correlate with poor relapse-free survival in independent TNBC patient datasets. In support of a tumor suppressor role, SNX3 protein levels are downregulated in breast cancer major subtypes in the Clinical Proteomic Tumor Analysis Consortium (CPTAC) data (Figure 1) [32].

Of note, SNX3 plays a role in recycling other receptors (e.g., transferrin receptor, WLS, and potentially other ErbB receptors). Indeed, transferrin receptor levels were also upregulated in SNX3-long-term silenced TNBC cells but not in primary tumors where EGFR was overexpressed. Our data suggest reduced recycling processes to activate and select dynamic and context-dependent compensatory mechanisms to repopulate receptor levels to maintain the TNBC phenotype. Notably, overexpression of EGFR upon long-term silencing of SNX3 may explain EGFR overexpression cases in breast tumors with no genomic amplification or mutations. The study’s results highlight intricate relationships between activated cell surface receptors, endocytosis-related proteins, and unclear transcriptional feedback mechanisms.

## CONCLUSIONS

Mutated or deregulated many endosomal trafficking proteins in cancers are linked to aberrant receptor trafficking, recycling, degradation, and duration of signaling during tumor progression and metastasis [3]. SNX3, an endosomal trafficking protein, is an emerging tumor suppressor in breast cancers as a target of the EGF-activated EGFR pathways and a modulator of EGFR protein levels.

In breast cancers, EGFR overexpression in all breast cancer subtypes is associated with poor clinical outcomes, large tumor size, and poor differentiation [33]. However, although EGFR is frequently overexpressed in half of the aggressive TNBCs and inflammatory breast cancers, *EGFR* gene amplification or activating mutations of *EGFR* are infrequent in breast cancers [34]. Because the underlying mechanisms of EGFR overexpression have not been well established in breast cancers, looking into potential feedback mechanisms related to endocytic processes may be helpful.

Overall, given the complexity of endocytic processes, the critical players and their regulation by post-translational modifications all add to the complexity of EGFR-activated signaling cascades. A better understanding of these backstage mechanisms will allow a more comprehensive understanding of receptor fate and activity. Finally, before we can consider key endocytosis regulators as therapeutic targets, these candidate proteins must also be evaluated within the context of potential feedback mechanisms to modulate the biosynthesis and repopulation of receptors in cancer cells.
